# Amphricrine carcinoma of the cervix-adeno neuroendocrine tumor: A case report

**DOI:** 10.4274/tjod.72325

**Published:** 2016-06-15

**Authors:** Erdem Fadıloğlu, Alper Karalök, Osman Türkmen, Şeyma Asiltürk, Reyhan Öçalan, Nurettin Boran, Heyecan Ökten, Taner Turan, Gökhan Tulunay

**Affiliations:** 1 Etlik Zübeyde Hanım Women’s Health Training and Research Hospital, Clinic of Gynecologic Oncology, Ankara, Turkey; 2 Etlik Zübeyde Hanım Women’s Health Training and Research Hospital, Clinic of Pathology, Ankara, Turkey

**Keywords:** Cervical carcinoma, amphicrine tumor, neuroendochrine tumor

## Abstract

Adenoneuroendocrine carcinoma is a very rare form of cervical carcinoma that includes both endocrine and exocrine components. In general terms, these carcinomas progress aggressively and show early metastases due to the neuroendocrine component. The most important criteria related to prognosis is the stage of the disease. Without clearly determined therapeutic protocols this carcinoma is generally seen at earlier ages and causes high mortality. Many radiotherapy and multidrug chemotherapy protocols are used after surgical intervention. Detection of the neuroendocrine component of cervical tumors is achieved through immunohistochemical staining. Herein, we present a woman aged 50 years who was admitted to the hospital with abdominal pain and postmenopausal vaginal bleeding whose examination revealed a cervical tumor. A pathologic examination after surgery resulted as “adenocarcinoma and large cell neuroendocrine carcinoma.” Afterwards, a combined chemotherapy regimen (cisplatin + etoposid) was administered to the patient and 6 months of progress is evaluated in this report.

## INTRODUCTION

Adenoneuroendocrine carcinoma includes both endocrine and exocrine components. The histopathologic classification of these tumors was defined by Lewin^(1)^ as listed below:

1. Mixed tumors (both tissues exist together),

2. Both tissues exist completely separately,

3. Tumors that exist of amphricrine cells.

These tumors may occur in the sinonasal cavity, larynx, lungs, gastrointestinal system, and uterine cervix. Neuroendocrine tumors account for one percent of all cervical carcinomas and are classified into three groups histopathologically; (i) carcinoid tumor, (ii) atypical carcinoid tumor, and (iii) high grade neuroendocrine carcinoma; small cell or large cell types^(2)^.

Early nodal and distant metastases are typical for these tumors. Between all pathologic groups, small-cell carcinomas show the worst prognosis and have similar survival to small cell carcinomas of the lungs. Compared with other cervical carcinoma histopathologies, distant metastases occur frequently and there are shorter survival periods in neuroendocrine carcinoma^(3)^. Due to the lack of data, there is no consensus on the definitive treatment of the disease. Surgery alone is not adequate because of the frequent metastases and aggressive behavior. Following surgery, adjuvant chemotherapy and radiotherapy present better survivals in patients with non-small cell disease^(4)^.

The definitive diagnosis of neuroendocrine tumors can be achieved through immunohistochemical staining. Epithelial membrane antigen (EMA), cytokeratin 7 (CK7), cytokeratin 19 (CK19), chromogranin, and synaptophysin staining are diagnostic. Polymerase chain reaction (PCR) study of these viruses is also crucial because of the strong relationship with human papillomavirus (HPV) type 16 and 18.

Cervical adenoneuroendocrine carcinoma can be very similar to adenocarcinoma of the cervix. The differential diagnosis is very important because of the aggressive behavior of these tumors and the need for a more aggressive treatment than with other cervix carcinomas.

## CASE REPORT

A woman aged 50 years who had been post-menopausal for 8 years was admitted to the hospital with abdominal pain and ongoing postmenopausal vaginal bleeding, which had lasted for ten days. A gynecologic examination revealed a 2x1.5-cm polypoid lesion that originated from the endocervical canal. Also, the whole vagina seemed fragile and the cervix seemed to fuse with the vagina, and the vaginal fornix appeared to be erased. A 3x4-cm cervical tumor was diagnosed. The left parametrium was shortened and there was suspected involvement seen in the right parametrium. Abdominal ultrasonography showed normal uterine fundus and corpus with an endometrial thickness of 15 mm. The right adnexal area included a solid lesion that was 89x50 mm in diameter with cystic areas, and a 72x74 mm complicated cystic mass was seen in the left adnexal area, which was adjacent to the other mass. Abdominopelvic tomography revealed increased dimensions of uterus and cervix with heterogeneous structure. There was minimal fluid in the endometrial cavity. The right adnexal area included a mass of 80 mm that could not be distinguished from the uterus and showed retention of contrast matter. This mass was regular-edged and hypo-dense. The left adnexal area included a 90 mm complicated cyst. The peritoneal surfaces had implants, the largest of which was 53 mm. Massive ascites was present. The right lobe of the liver had 15 and 20 mm lesions that retained contrast matter.

The tumor marker results were as follows; CA-125: 1870 IU/mL, CA 19-9: 234 IU/mL, CA 15-3: 36.7 IU/mL, and carcinoembryonic antigen: 1.98 IU/mL. A cervical and endometrial biopsy were performed and both results were reported as adenocarcinoma. Accordingly, a laparotomy with a midline incision was performed. Nearly 7000 mL of serous ascites was explored. Adnexal masses were explored as they had been imaged on ultrasonography and tomography. Tumoral implants were seen on the peritoneum of the douglas pouch, hepatorenal area and paracolic area, serosa of rectosigmoid colon, and mesothelium of the bowel. The pancreas and spleen were normal and omental cake was observed. Right unilateral salpingo-oophorectomy was performed and frozen sections were reported as “poorly differentiated adenocarcinoma of the cervix.” As a result, surgery was expanded to type 2 radical hysterectomy, left unilateral salpingo-oophorectomy, resection of the sigmoid colon, right hepatorenal peritoneum, and tumoral implants both on the liver and mesothelium of bowel and appendectomy. Right diaphragm stripping was performed due to the tumoral implants of the right diaphragm. Bilateral pelvic and para-aortic complete lymphadenectomy was performed until the level of left renal vein. The operation was completed with optimal cytoreduction, which resulted with the existence only of residual tumor with a greatest diameter of 5 mm.

### Pathology

**Macroscopic:** The first examined tissue was frozen sections of right salpingo-oophorectomy. The tumoral lesion was a solid structure with minimal cystic structures within, with white-yellowish color and necrosis. Ovarian and tubal tissues were had tumoral nodules on their surface.

**Microscopic:** After fixation with 10% formalin and staining with Hematoxylin and Eosin on paraffin sections, two different malignant tissue was remarkable on cervix. The first tumor was characterized with pseudo-stratified prismatic epithelium with hyperchromatic nuclei and different diameters of atypical glands with mitosis. These atypical glands were at a random distribution both at mucinous stroma and desmoplastic stroma.

**Figure 1:**
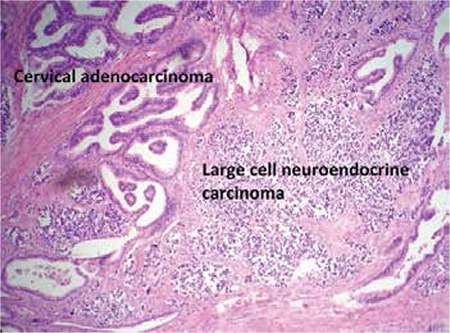
Mix cervical adenocarcinoma and large cell neuroendocrine carcinoma

The second tumor comprised large cells with atypical nuclei and significant nucleoli. There were a high number of mitotic cells forming trabecular, cordon-like structures with a palisade sequence. Necrosis and lymphovascular invasion were also noticed (Figure 1).

More than 50% percent of cervical muscle tissue was invaded with tumoral tissue. The vagina, both parametrium, uterine and cervical peritoneum were also showing invasion. The endometrium was inactive and only adenomyosis was noticeable in the myometrium.

**Figure 2:**
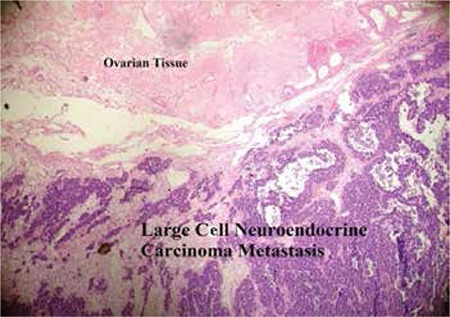
Large cell neuroendocrine carcinoma (ovarian metastasis)

The tumoral tissue on the right ovary was also showing characteristics of the second tumor described above. The cystic structure on left ovary and tumoral tissues on both tuba uterine, serosa of the recto-sigmoid colon and adjacent adipose tissue were showing the same structural features (Figure 2).

Immunohistochemical Study: For the definitive diagnosis, proper immunohistochemical tests were performed. The tumoral tissue on right ovary was stained diffusely positive with EMA, CK7, chromogranin, synaptophysin, focally positive for progesterone receptor, and negative for estrogen receptor, thyroid transcription factor-1 (TTF-1), CK, gross cystic disease fluid protein 15 (GCDFP15), CK K-20, CD-56. These results were compatible; both the ovarian and cervical tumors were large cell neuroendocrine carcinoma. HPV PCR studies also revealed HPV type 16 positivity for both tumors. The Ki 67 proliferation index was 80%. According to all of these data, the ovarian tumor was a metastasis of cervical neuroendocrine carcinoma. The definitive pathologic diagnosis was reported as mixed adenoneuroendocrine carcinoma of the cervix (mucinous adenocarcinoma, endocervical type + large cell neuroendocrine carcinoma). The stage of disease was 4B according to 2009 International Federation of Gynecology and Obstetrics criteria.

The other tumoral implants were metastasis of the cervical primary tumor. Also 1 right hypogastric and 1 presacral lymph node were infiltrated with tumor. Other lymph nodes and abdominal cytology were clear.

After the operation, 6 cycles of etoposide + cisplatin combined chemotherapy were given with 2-week intervals. During that period, abdominal and thoracic tomography showed no new tumor lesions. Tumor markers 6 months after the surgery were: Carcinoembryonic antigen: 0.30 ng/mL, CA 15-3: 10.6 IU/mL, and CA 125: 14.3 IU/mL.

## DISCUSSION

Cervical adenoneuroendocrine carcinomas are extremely rare. These malignancies progress aggressively with distant metastasis and high mortality rates. There is no consensus on suitable therapeutic protocols because of the lack of data^(5)^. According to the available data, the most important prognostic factor is the stage of the disease^(6)^. Studies reflecting data about small-cell neuroendocrine carcinoma suggest multidrug aggressive chemotherapeutic protocols including cisplatin and etoposide^(7)^. Despite the higher stage of the presented case, 6 months of follow-up showed a very good prognosis with surgery and cisplatin + etoposide chemotherapy regimen.

One of the most important prognostic features of adenoneuroendocrine carcinomas is that the malignant potential is mostly related to the neuroendocrine component. The presented case was consistent with this and the metastases originated from neuroendocrine component. Despite these data, ovarian metastasis may rarely originate from lower grade adenocarcinoma components and the differential diagnosis of these malignancies with primary ovarian carcinomas can be made through HPV DNA positivity^(8)^.

An association between cervical neuroendocrine carcinomas and high-risk HPV (type 16 and 18) has been described^(9)^. HPV produces E6 oncoprotein, which causes the demolition of p53 rather than the effects on retinoblastoma^(10)^. Another molecular mechanism is the loss of heterozygosity on 3p loci as seen in the pathophysiology of small-cell lung cancers^(11)^. Considering all of these pathways, it may be speculated that the malignant transformation of a cervical cell is a multifactorial process.

Another point is that neuroendocrine carcinomas may cause paraneoplastic syndromes. No paraneoplastic syndromes were observed in our follow-up, but different syndromes have been experienced^(12)^.

## CONCLUSION

Cervical adenoneuroendocrine carcinoma is a very rare form of cervical malignancies and there is no consensus for proper treatment. The prognosis is mostly related to neuroendocrine component. The most important prognostic factor is indicated as the stage of the disease. The association between neuroendocrine malignancies and HPV subtypes are clinically important for diagnosis. It must be highlighted that differential diagnosis between adenocarcinoma and adenoneuroendocrine carcinomas may be challenging, but is essential because of the different clinical approach and prognosis. Immunohistochemistry is widely used in the differential diagnosis. More extended data should be presented to provide for definitive treatment protocols and clinical approaches.
